# The Burden of Comorbidities in Obstructive Sleep Apnea and the Pathophysiologic Mechanisms and Effects of CPAP

**DOI:** 10.3390/clockssleep5020025

**Published:** 2023-06-19

**Authors:** Victoria Sircu, Silvia-Iaroslava Colesnic, Serghei Covantsev, Olga Corlateanu, Anna Sukhotko, Cristian Popovici, Alexandru Corlateanu

**Affiliations:** 1 Division of Pneumology and Allergology, Department of Internal Medicine, State University of Medicine and Pharmacy Nicolae Testemitanu, MD-2004 Chisinau, Moldova; sircu.victoria@yahoo.com (V.S.); aroslavakolesnik@gmail.com (S.-I.C.); cris.popovici9@gmail.com (C.P.); 2 Department of Clinical Research and Development, Botkin Hospital, 125284 Moscow, Russia; kovantsev.s.d@gmail.com; 3 Department of Emergency Medicine № 76, Botkin Hospital, 125284 Moscow, Russia; 4 Department of Internal Medicine, State University of Medicine and Pharmacy Nicolae Testemitanu, MD-2004 Chisinau, Moldova; olga.corlateanu@usmf.md; 5 Department of General Oncology № 71, Botkin Hospital, 125284 Moscow, Russia; syxotya26@yandex.ru

**Keywords:** OSAS, CPAP, diabetes mellitus, hypertension, ischemic heart disease, stroke, renal failure

## Abstract

Micro-arousals and the repeated desaturation of oxyhemoglobin, which are typical in obstructive sleep apnea syndrome (OSAS), have adverse effects on the health of patients, leading to a wide range of complications such as cardiovascular (arterial hypertension, pulmonary hypertension, chronic heart failure, arrhythmias, myocardial infarction), cerebrovascular (strokes), metabolic (insulin resistance, obesity, diabetes mellitus, metabolic syndrome), gastrointestinal (non-alcoholic liver disease), urinary (chronic renal failure), and neuropsychiatric complications as well as a wide range of malignancies. These, in turn, have multilateral effects on familial, occupational, and social life, as well as increasing the risks of road traffic accidents and accidents at the workplace. Awareness, timely screening, and the prevention of complications play important roles in diagnosing and treating comorbid conditions. This review focuses on comorbidities in OSAS and the effect of Continuous Positive Airway Pressure (CPAP) therapy on their prognoses.

## 1. Introduction

Obstructive sleep apnea syndrome (OSAS) is one of the most important disorders discovered in the last 50 years because of its particular impact on all organs and systems as well as its reduction of the quality of life [1]. The repeated desaturation of oxyhemoglobin and micro-arousals, which are typical in obstructive sleep apnea syndrome (OSAS), have a negative impact on the health of patients, leading to complications from the cardiovascular system—arterial hypertension, pulmonary hypertension, chronic heart failure, arrhythmias, myocardial infarction, stroke, insulin resistance, diabetes mellitus, metabolic syndrome, and chronic renal failure—and neuropsychiatric complications (depression, irritability, low level of attention, loss of short-term memory, etc.), which affect familial, occupational, and social life, as well as increasing the risk of road traffic accidents or accidents at the workplace [1]. The particular importance of OSAS is associated with an increase of its incidence by 14–55% depending on age and gender [1]. Such an increase has great importance for the healthcare system and points to the need for timely and effective screening [2].

A significant impact in the development of complications is obesity, which is a major risk factor. Influence by the hypoxia adipocytes leads to changes in adipocytokine secretion that contribute to insulin resistance and metabolic syndrome in patients with OSAS. Intermittent hypoxia also causes the decline and necrosis of pancreatic beta cells because of oxidative stress [1].

Adipose tissue inflammation and local hypoxia contribute to increased cytokine levels, oxygen-free radicals, tumor necrosis factor-alpha, pre-atherogenic chemokines, and proangiogenic peptides, some of which lead to the activation of the sympathetic nervous system with endothelial dysfunction, arterial rigidity, and atherosclerosis [3]. Sympathetic afferents generate renin-angiotensin system activation and *hydrosaline metabolism modification,* which, combined with the reduction of baroreceptor sensitivity, results in the development of arterial hypertension (AH) [4]. Studies have demonstrated an association of OSAS with hypercoagulation and decreased fibrinolytic activity that results in a prothrombotic status with an increased risk of thrombotic complications [5]. Men with an apnea/hypopnea index (AHI) of more than 19 and women with an AHI of more than 25 are at significantly higher risk of stroke than a healthy person [6]. An equally important effect of hypoxia is the proinflammatory state, which is associated with systemic inflammatory response syndrome and oxidative stress [7]. Based on the above, we noted the particular importance, in the diagnosis of OSAS, of the critical role played by comorbidities. The late detection and treatment of comorbidities can lead to severe and dangerous complications.

## 2. Results

### 2.1. Cardiovascular Diseases

At the moment, there is not only circumstantial evidence that specifies the role of OSAS in the etiology and progression of cardiovascular diseases, especially high blood pressure (HBP), but also direct evidence gathered over the past decade [1]. One of the reasons for this phenomenon may be the neglect of OSAS evaluation in many previous epidemiological studies [8]. To a certain extent, this neglect was attributable to the high costs that are required for OSAS detection in large samples of the population. Additionally, OSAS patients often suffer from concomitant diseases such as obesity, HBP, diabetes mellitus, chronic obstructive pulmonary disease (COPD), bronchial asthma, and glucose intolerance status; therefore, any independent effect of OSAS on cardiovascular risk could be masked under comorbidities.

However, some prospective research papers aimed at investigating the incidence of cardiovascular diseases, and assessment studies of the therapeutical effect of CPAP, have provided accurate and indisputable evidence, confirming the close cause–effect relationship between OSAS and cardiovascular pathology [1].

### 2.2. Hypertension

The most conclusive evidence confirming the role of OSAS in HBP occurrence is derived from the well-known studies involving the Wisconsin Sleep Cohort [9]. In the studied population re-evaluated 4 years after the initial investigation, apnea–hypopnea index rates higher than 15 events per hour, regardless of other factors, were associated with an increase by 3 times of risk of HBP development [10]. HBP incidence in OSAS patients is approximately 30–70% [11]. Usually, the incidence of HBP and resistant hypertension increases with OSAS aggravation [1].

These data suggest that a significant proportion of cases that were previously considered essential hypertension may reflect consequences of undiagnosed and, as a result, untreated OSAS. Consensus guides on the management of hypertension reflect the increasing amount of evidence of OSAS involvement in HBP etiopathogenesis. In 1997, the sixth Report of the Joint National Committee on Prevention, Detection, Evaluation, and Treatment of High Blood Pressure, which, for the first time, reported the critically important role of OSAS, also recommended the exclusion of this pathology in the assessment of HBP causation [12]. Further, guides published in 2003 placed OSAS in the top of the identified causes of resistant hypertension [13]. Additionally, other guides published between 2017–2018 confirmed the importance of OSAS in HBP management [14].

CPAP therapy for obstructive sleep apnea syndrome significantly reduces diurnal blood pressure, not only in patients with resistant hypertension [1] but also for patients with relatively mild forms of arterial hypertension [1].

Even if the effect of blood pressure reduction is not apparent in normotensive patients with OSAS on long-term CPAP treatment [15], two placebo-controlled randomized trials, in which placebo was CPAP at sub-therapeutic dose, have demonstrated that an extended duration of therapy resulted in slight, but statistically significant, decreases of daily blood pressure from 1.3 to 5.3 mm Hg [1]. Therefore, there is ample evidence reaffirming the role of untreated OSAS in the etiopathogenesis of HBP development; moreover, studies demonstrate a significant reduction in diurnal blood pressure for CPAP in patients [16].

### 2.3. Myocardial Ischemia

OSAS induces different types of stress, chronic and acute, that may predispose patients to sleep-related myocardial ischemia. In conditions of pronounced acute hypoxemia and CO2 retention, the activation of the sympathetic nervous system and dramatic blood pressure elevation can trigger myocardial ischemia. In a recent study, researchers found a possible link between bridging night-shift work, increased levels of inflammatory markers, and carotid intimal medial thickness [17]. In the long term, the establishment of diurnal hypertension and the increased production of vasoactive and trophic substances (such as endothelin), along with the activation of proinflammatory and procoagulant mechanisms, can also contribute to the development and progression of ischemic heart disease (IHD). In the *Sleep Heart Health Study cohort*, OSAS was recognized as an independent risk factor for IHD [18]. Nocturnal changes of the ST segment, confirming myocardial ischemia, were found in patients with OSAS with no clinical signs of IHD [19]. ST segment depression occurs more frequently in patients with a severe form of OSAS who have a history of nocturnal angina pectoris symptoms and depends on arterial oxygen saturation [20]. CPAP treatment considerably reduces the overall duration of ST segment depression in patients with sleep apnea [21]. In addition, some epidemiological studies have confirmed the association between OSAS or snoring with myocardial infarction (MI) [22]. 

Obstructive sleep apnea is common in patients with MI in their histories [23]. Postinfarction modifications in cardiac function may predispose patients to sleep apnea development or the impairment of previously diagnosed OSAS. At the same time, for patients with IHD, obstructive sleep apnea may constitute a prognostic predictor. The monitoring of 62 patients with detected IHD for a duration of 5 years identified a high mortality rate (38%) in the OSAS group compared to the non-OSAS patient group, taking into consideration other influential factors [24].

### 2.4. Cardiac Rhythm Disorders

Heart rhythm disorders occur in approximately 18–48% of OSAS patients, although it is difficult to evaluate their actual prevalence because of the limited number of groups included in research and the considerable number of different types of arrhythmias [1]. The presence of complicated tachyarrhythmias and bradyarrhythmias increases the risk of cardiovascular complications, reduces the quality of life, and increases the risk of unfavorable outcomes [25]. Nocturnal oxygen desaturation is an independent risk factor for the development of atrial fibrillation [1]. The presence of OSAS is also a risk factor for atrial fibrillation recurrence after successful cardioversion [1]. However, in one randomized trial that compared the patients on CPAP and non-CPAP therapies, no significant difference was observed in the frequencies of arrhythmias between the groups [1]. The prevalence of bradyarrhythmias is about 8% in patients with AHIs of less than 60, compared to 20% in patients with AHIs of more than 60 [26].

Ventricular arrhythmias occur in about 5% of the general population, whereas it has been found in 14–74% of patients with OSAS [27]. The prevalence depends on the condition of AHI and desaturation being below 90% [28]. Moreover, it should be noted that 60% of patients with ventricular arrhythmias hospitalized for catheter ablation or cardioverter-defibrillator implantation had AHIs of more than 5, and 34% had moderate-to-severe stages of OSAS [1].

Considering the diversity of arrhythmias that can occur in OSAS patients, it is difficult to evaluate their impact on a patient. Most probably, short episodes of bradycardia may not be of significant importance, whereas atrial fibrillation and ventricular rhythm disorder are the severe risk factors for thromboembolic events and sudden death [27].

**Figure 1:**
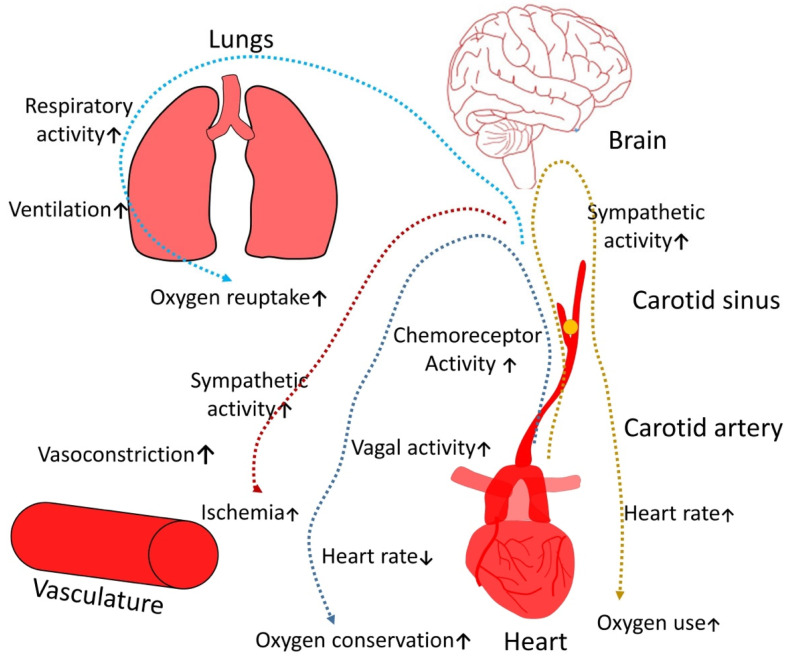
Pathogenetic role of OSAS on the symphato-vagal balance (yellow arrow—sympathetic system activity; red arrow—vascular aches; light blue arrow—respiratory changes; violet arrow—parasympathetic activity). ↑ arrow—increased activity. ↓ arrow—decreased activity.

As a result of upper airway obstruction, the proportion of blood gases changes, leading to hypercapnia and hypoxemia. In these conditions, respiratory and cardiovascular activity responses change through central and peripheral control mechanisms. Hypercapnia is one of the most important triggers for the respiratory brain center. It leads to increased ventilation and oxygen reuptake and also causes increased sympathetic activity, enhancing oxygen intake, which ultimately leads to ischemia; another crucial factor is the provision by hypoxemia of a stimulatory effect on a vagal tone that significantly increases the risk of conduction rhythm disorders and bradycardia (Figure 1).

**Figure 2:**
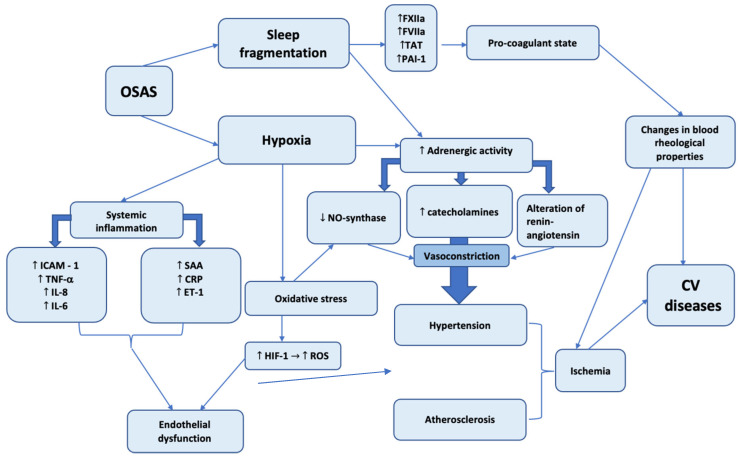
Pathogenetic mechanisms of OSAS on cardiovascular disease development. ↑ arrow—increased activity. ↓ arrow—decreased activity.

The presented scheme demonstrates the two most important pathogenetic mechanisms caused by OSAS: hypoxia and sleep fragmentation. The role of hypoxia in the initiation and progression of diverse pathological conditions cannot be underestimated. The cardiovascular system is affected by systemic inflammation, oxidative stress, and adrenergic activity. Both systemic inflammation and oxidative stress induce the synthesis of different factors, leading to endothelial dysfunction, which is closely related to atheromatosis—an essential element of coronary artery disease (Figure 2). The oxidative stress promotes atherosclerosis through multiple mechanisms—lipid oxidation, DNA oxidation, and endothelial dysfunction—and the last of these in turn leads to maladaptive changes of endothelium and is strongly associated with the progressive development of atherosclerosis.

Hypoxia and sleep fragmentation action are associated with increased adrenergic activity, which mediates vasoconstriction and the development of hypertension.

At the same time, sleep fragmentation is correlated with increased synthesis of blood clotting factors and the deterioration of hemorheological properties. The total action of these factors induces the development and progression of diverse cardiovascular diseases.

### 2.5. Neurophyschiatric Deviations

Neurocognitive disorders associated with OSAS include daytime sleepiness, poor concentration, depression, and even dementia [29].

Alzheimer’s disease (AD) is the most common form of dementia, and its prevalence increases with age. Several studies have demonstrated that cognitive disorders occur more frequently in OSAS patients [30]. A recent meta-analysis showed that about 50% of AD patients are encountered by OSAS after their primary disease diagnosis [30].

There is a lot of evidence that suggests OSAS influences on AD progression, sleep fragmentation, intermittent hypoxia, and hemodynamic changes may induce a cumulative effect on Alzheimer’s disease development, and this suggests that timely and sufficient CPAP therapy may help to prevent or to reduce cognitive decline and dementia [31].

Studies in neurodegenerative disorders have demonstrated that OSAS has also been associated with an increased risk of Parkinson disease development [31,32].

### 2.6. Cerebrovascular Pathology

The presence of snoring predisposes patients to an increased risk of stroke, regardless of other cardiovascular risk factors. It has also been found that sleep apnea has an increased prevalence in patients with stroke [33], but it is still unknown whether sleep apnea is an independent risk factor for cerebrovascular diseases. Hemodynamic, vascular, inflammatory, and thrombocytic pathogenetic factors that are activated in OSAS can lead to an increased risk of cerebrovascular disease development, regardless of the circumstances. Acute episodes of apnea lead to dramatic falls in cerebral blood flow [34]. Ischemia is induced by repeated episodes of sleep apnea intensified by associated hypoxia, as well as any pre-existing modifications of autoregulation or vasodilator reserves. Thus, OSAS, directly or indirectly and through concomitant diseases, increases the risk of stroke. At the same time, stroke can trigger respiratory disorders during sleep: central and obstructive apnea [35]. CPAP therapy plays an important role in the therapy of OSAS comorbidities. A significant improvement of collateral cerebral blood flow was observed in patients with OSAS on long-term CPAP therapy [36].

Some authors have indicated an increased frequency of OSAS occurrence among patients with stroke. Among the patients from the stroke department, 73.7–86% had AHIs of over 5, and about a third had AHIs of over 30 [37,38].

However, the relationship between embolic stroke and OSAS is still indirect. Further long-term works are needed to establish whether OSAS an important cause of cerebrovascular diseases independent of other factors. Considering that OSAS is a modifiable risk factor for cerebrovascular diseases, specialists need to pay particular attention to this [39].

### 2.7. Peripheral Neuropathy

Chronic oxygen deprivation can lead to both central and peripheral nerve injury [40]. Patients with OSAS often have nerve dysfunction, the severity of which is partly related to the level of nocturnal hypoxemia. Current studies demonstrate that abnormal nerve conduction suggests axonal lesions and demyelinating neuropathies [41]. Clinical signs of polyneuropathy can be seen in up to 71% of patients with OSAS. The severity of axonal damage tends to correlate with the percentage of night time with an O_2_ saturation of below 90% [42]. Moreover, the risk of polyneuropathy increases in the case of other comorbidities such as diabetes [43]. This damage, at least to some degree, can be reversible with proper CPAP treatment for sleep apnea [44].

### 2.8. Depression

Among patients with OSAS, depression was found in 5–63% of cases. At the same time, it should be noted that many symptoms of these pathologies are similar. Sleep disorders are rarely studied in patients with depressive disorder, and depression is rarely evaluated in patients with OSAS. The bidirectional interaction of these two conditions is complicated and should be closely studied in the future [45]. The early screening of depressive disorder in patients with OSAS can lead to the timely psychological and social rehabilitation of these patients [46].

Nevertheless, a large cohort study, which was held from 1991 to 2015 and included 10149 patients over a median follow-up of 9.7 years, showed no correlation between OSAS and depression [47]. Some of the depression cases in patients with OSAS may be results of other factors such as biological (i.e., other diseases) and social (i.e., unemployment, family conflicts, and so on) factors. It is important to note that the gravity of OSAS, obesity, and gender are significant factors that need to be considered for the precise determination of the real cause of depressive disorder [48].

Creating awareness, the timely screening of both depression and OSAS, and the consideration of a possible interaction between these two disorders is an essential step in combating both illnesses [49]. This fact also highlights that since these diseases are characterized by “masks”, a correct diagnosis and treatment requires a multidisciplinary team of specialists that includes a clinical psychologist or psychiatrist.

### 2.9. Obesity

Weight gain is a slow and multifactorial process associated with lifestyle factors such as short sleep duration, sedentary lifestyle, excessive caloric intake, and genetics. It is estimated that approximately 40% of patients with a body mass index of greater than 28 suffer from OSAS, with a tendency towards higher morbidity simultaneously appearing with weight gain [50].

Short sleep duration and higher caloric intake can cause hormonal imbalances. One such imbalance is a reduced level of melatonin, which leads to changes in the metabolic circadian rhythm, predisposing patients to weight gain and metabolic alterations [51].

There are also leptin and insulin modifications; it has been demonstrated that an obese person develops resistance to both leptin and insulin. Leptin, which physiologically reduces appetite and accelerates energy metabolism, was found to be decreased in patients with short sleep durations, and this in turn had increased their appetite and led to weight gain, but more than this, it was demonstrated that ghrelin, which stimulates appetite, was elevated in people who had short sleep durations [52]. Furthermore, a positive impact on OSAS has also been demonstrated in patients after bariatric procedures and sleeve gastrectomy, being characterised by the resolution of or improvement in OSAS [53,54].

### 2.10. Gastrointestinal Disease

Recent studies have demonstrated that sleep deprivation and impaired sleep quality are associated with various gastrointestinal disorders. The true nature of these changes is complicated, but it is tightly linked to metabolic changes, proinflammatory cytokines, and gut microbiota. Altogether these factors can cause a systemic reaction in an organism, not being limited only to the gastrointestinal tract [55,56]. Approximately 10% of patients with snoring or OSA have revealed that functional dyspepsia is associated with more severe daytime sleepiness and higher apnea–hypopnea indices compared to those without functional dyspepsia [57]

In a cross-sectional study of 5792 subjects that were surveyed as part of a community-based cohort, the subjects provided information regarding their quality of sleep, according to the Pittsburgh Sleep Quality Index (PSQI), and digestive symptoms, as assessed by the Gastrointestinal Symptom Rating Scale (GSRS). The results revealed that sleep disturbances were associated with digestive symptoms (aOR = 1.29, 95% CI = 1.22–1.36), especially abdominal pains (aOR = 1.63, 95% CI = 1.19–2.25), acid regurgitation (aOR = 1.48, 95% CI = 1.17–1.86), abdominal distension (aOR = 1.80, 95% CI = 1.42–2.28), and eructation (aOR = 1.59, 95% CI = 1.24–2.03) [58]. This study demonstrated a tight link between sleep quality and gastrointestinal diseases. Similar studies have demonstrated an increased risk of inflammatory bowel disease. The odds ratio of IBS in a positive sleep apnea group versus in a negative sleep apnea group were found to be 3.92 (95% confidence interval = 1.58–9.77, *p* = 0.003) [59].

### 2.11. Nonalcoholic Fatty Liver Disease

NAFLD is characterized by the excessive accumulation of lipids in hepatocytes, which results in the lipotoxicity and inflammatory damage of hepatocytes.

Intermittent hypoxia leads to tissue hypoxia and can result in oxidative stress, mitochondrial dysfunction, inflammation, and increased sympathetic nervous system activity. In studies on these phenomena, intermittent hypoxia has been associated with insulin resistance—a key factor of hepatic lipid metabolism dysfunction, hepatic steatosis, and fibrosis, each of which is involved in the development and/or progression of NAFLD [54,60].

In a study, Pretta et al. found an independent association between nocturnal oxygen saturation values and significant liver fibrosis in adult patients’ biopsy results; severe NAFLD was spotted a low prevalence of morbid obesity [61]. Moreover, several studies have reported significant improvement in AST, ALT, and ALP levels in patients after 6 months of CPAP therapy [62,63,64].

### 2.12. Diabetes Mellitus

The prevalence of diabetes mellitus among OSAS patients is about 23–48% [65,66]. Experimental studies have shown that sleep restriction to 4 h per night for six nights is associated with impaired glucose tolerance [67]. The data obtained from one study found that young and healthy night shift workers show increased expression of leukocyte interleukin-1β RNA and a significant correlation of IL-1β expression with HbA1c blood levels [68]. Another important mechanism of diabetes development in OSAS patients is decreased insulin secretion, which leads to short-term or long-term hyperglycemia [69]. Moreover, OSAS is associated with low adiponectin levels and insulin tolerance and the elevation of cortisol and catecholamines [70,71]. It has been observed that during fasting, glycated hemoglobin and blood glucose levels are correlated with AHI, sleep duration, and oxygen saturation of lower than 90% [72,73].

In conditions of proinflammatory state and oxidative stress, special attention should be paid to the methods with potential to improve metabolism and reduce the negative impact of hypoxia. After six months of CPAP treatment, a decrease in endothelial dysfunction, inflammatory mediators, and lipid peroxides has been observed [74,75]. The results of CPAP influence on glucose metabolism and insulin resistance are still controversial. Several researchers have observed improvements in glycated hemoglobin levels and insulin sensitivity in nondiabetic patients [76]. J. F. Guest et al. observed similar results in patients with type 2 diabetes and OSAS [77]. Furthermore, it has been demonstrated that the incidence of type 2 diabetes is reduced in OSAS patients on regular CPAP therapy compared to non-CPAP patients [78]. However, some studies have not confirmed the positive effect of CPAP on the glycemic profile in patients on CPAP therapy [79].

**Figure 3:**
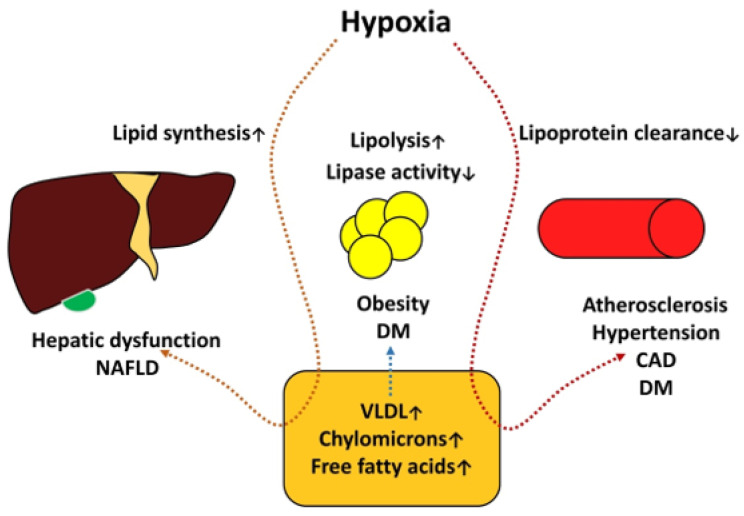
The pathogenesis of metabolic disorders and OSAS. ↑ arrow—increased production/activity. ↓ arrow—decreased activity.

The pathogenesis of metabolic disorders and OSAS is summarized in Figure 3.

Diabetes mellitus, obesity, and non-alcoholic fatty liver disease constitute components of metabolic syndrome. Insulin and leptin resistance and elevated levels of ghrelin, mediated by short sleep duration, have been associated with sleep restrictions and increased catecholamine and cortisol levels, and hypoxia can amplify hyperglycemia, negatively affecting these processes. This situation shows the important roles of insulin resistance and non-alcoholic fatty liver disease. In these conditions, the regulatory capacity of insulin on hepatic lipase is compromised and the situation becomes complicated by hypoxia. Hypoxia induces hepatocyte injury with hepatic lipid metabolism alteration; specifically, it increases lipid synthesis and causes a buildup of fat in the liver, decreasing lipase activity and increasing lipid synthesis, leading to alterations of the lipid profile that are essential for endothelial dysfunction—a crucial factor of atherogenesis.

### 2.13. Chronic Kidney Disease

End-stage renal failure affects 57% of patients with OSAS [80,81]. Hypoxia, fluid retention, and rennin-angiotensin system activation are the key elements of the interconnection between OSAS and kidney failure, aggravating both conditions [82,83]. Nevertheless, not only can OSAS lead to kidney failure installation, but there is an inverse variant of this relation [84]. Due to the fact that OSAS patients often have comorbidities, such as AH, advanced atherosclerosis, and diabetes mellitus, there is a perception that chronic renal failure appears in the background of these illnesses. Nevertheless, in patients with CKD and diabetes, OSAS seems to be a factor that results in a higher urinary albumin–creatinine ratio and a lower estimated glomerular filtration rate [85]. OSAS is also a significant risk factor for mortality in dialysis patients and is by itself linked to metabolic disturbances, proteinuria, and arterial disease [86,87]. Preliminary data have indicated that CPAP therapy contributes to kidney hypoxia injury protection; however, further large-scale randomized trials are needed to estimate this effect [88]. Furthermore, up to 73% of patients with OSAS have kidney dysfunction, which is revealed during screening and brings up the importance of a multidisciplinary approach to this problematic group of multimorbid patients [89].

### 2.14. Malignant Neoplasms

As a result of intermittent hypoxia and sleep fragmentation, OSAS may be involved with cancer progression and, probably, with cancerogenesis. The reduced antitumor activity and enhanced immunosuppression were found as a result of an experimental cell-culture and mice-model study [90]. Short sleep duration was found to be associated with alterations in tumor-associated macrophage (TAM) phenotypes, specifically higher TLR4 expression, which plays an important role in tumor progression [91]; moreover, an experimental animal model of colon cancer study determined a certain relationship between the impact of sleep fragmentation and ROS-induced DNA damage, which in turn leads to cancerogenesis [92].

Intermittent hypoxia can also precipitate tumor growth and aggression through (1) hypoxia-inducible factor 1 (HIF-1) activation with angiogenesis and the stimulation of both tumor growth as well as metastatic rate [93], and (2) immune response changes, specifically those caused by the activation of *tumor-associated* macrophages [94]. The involvement of IH in cancerogenesis can be explained by oxidative stress induction and DNA oxidation with the creation of gene mutations and cell malignancies [95]. Sleep fragmentation, which occurs in OSAS, has been associated with high risk of cancerogenesis [96,97]. However, such data have mainly been received from studies on animals and cell cultures, where it is relatively easy to take into account such cofactors as age, obesity, and sleep time. All of these factors independently increase the risk of oncological disease development and, at the same time, are traditionally associated with OSAS [98].

Several major studies have found a relationship between OSAS and elevated risk of cancer development. The overall time with less-than-90% oxygen saturation has been associated with a 2.33-times increased risk of cancer development [99]. Similar results have been reported in patients from the prospective 20-year follow-up research by the same authors [100]. Nevertheless, age is one of the risk factors for cancer development. Another important point is the correlation of OSAS with different oncologic diseases. For instance, patients with OSAS have a 1.5 times-increased risk of CNS neoplasm in comparison with patients without apnea [101]. A large multicentric study that included 33711 patients demonstrated that while controlling for confounders, severe OSAS was associated with a 15% increased hazard of developing cancer compared with no OSAS (HR = 1.15, 1.02–1.30; ARD = 1.28%, 0.20–2.37; NNH = 78), and severe hypoxemia was associated with about a 30% increased hazard (HR = 1.32, 1.08–1.61; ARD = 2.38%, 0.47–4.31; NNH = 42) [102]. The relationship in question seems to depend on the type of cancer and severity of OSAS. Some cancers are not encountered as frequently as others in OSAS patients [103]. The differences between cancers in OSAS patients are presented in the table below Table 1. It seems logical that different tumors tend to react differently to oxygen deprivation. The results of some types of cancer are perplexing, and it is early to say whether there is a relationship between them and OSAS.

Some types of cancers are reported more frequently, particularly melanoma, bladder, lung, liver, cervix and kidney, and pancreas cancers. Moreover, it is too early to say whether the presence of OSAS can be related to an increased risk for metastasis or death [105].

Research into different oncological diseases in the context of hypoxia is especially important, both for the better understanding of the mechanisms of cancer development and for the selection of patient groups who need the timely screening and detection of OSAS.

**Figure 4:**
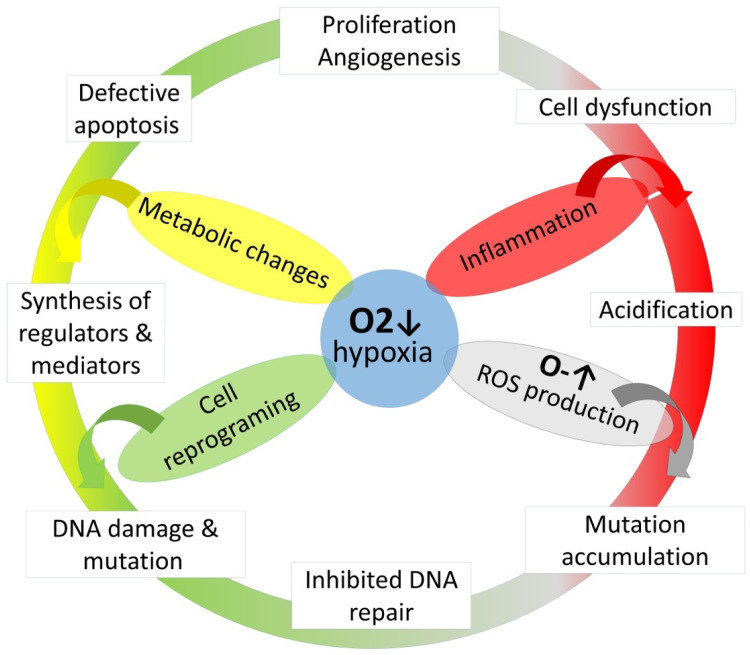
The role of hypoxia on cancerogenesis. ↑ arrow—increased oxygen levels. ↓ arrow—decreased oxygen levels.

Due to exposure to hypoxia, there are different pathological changes at the cellular level. Metabolic changes induce the synthesis of diverse regulators and mediators, which induce inflammation, cell dysfunction, and defective apoptosis. The oxidative stress provides DNA oxidation damage that leads to the inhibition of DNA repair and mutation accumulation, mediates the transformation of malignant cells, and also promotes the growth, proliferation, and invasion of malignant cells (Figure 4).

## 3. Conclusions

OSAS is one of the most important diseases discovered in the last 50 years. The accumulated knowledge has helped us understand that this pathology is associated with a marked function disorder not only of the respiratory system but also of many other systems. Due to hypoxia, proinflammatory syndrome, oxidative stress, and other processes, the dysfunctions of the cardiovascular, nervous, endocrine, excretory, and other systems have been observed. The timely screening of OSAS and CPAP therapy administration contribute to reparation and, in some cases, the marked deceleration of comorbidity progression. The modern approach to OSAS requires a multidisciplinary team that is able not only to reach correct diagnoses and treatment plans, but also to make adjustments according to the present comorbidities.

## Figures and Tables

**Table 1:** Association between OSAS and some types of malignant neoplasms.

Malignant Neoplasia	Possible Risk
CNS neoplasms	The overall risk for developing primary CNS cancers was found to be significantly higher in the OSAS group (aHR, 1.54; *p* = 0.046) after adjusting for age, gender, and obesity among other variables. Subgroup analysis revealed a significantly higher risk for primary brain cancers but not for primary spinal cord cancers [101].
Lung cancer	The data on lung cancer differs from study to study. Kendzerska and coworkers have reported a higher risk of developing lung cancer in a subgroup of OSAS patients with AHI Q4 vs. Q1 (1.78 [1.03–3.10] [102]. Sillah and coworkers have reported a protective effect of OSAS on the lungs (SIR 0.66, 95% CI 0.54–0.79) [104].
Melanoma	The risk of melanoma tends to increase with more severe AHI 2.49 (1.03–6.05) AHI: Q4 vs. Q1 [102]. Other studies have also demonstrated an increased risk of melanoma (HR = 1.13, CI = 1.09–1.18 and SIR 1.71, 95% CI 1.42–2.03) [105].
Breast cancer	The aHR of breast cancer in patients with OSAS was found to be higher [HR, 2.09; 95% confidence interval (CI), 1.06–4.12; *p* < 0.05] than that of the controls during a 5-year follow-up. Despite not meeting statistical significance, the authors reported an increase in the risk of breast cancer in women aged 30–59 years (HR, 2.06; 95% CI, 0.90–4.70) and ≥60 years (HR, 3.05; 95% CI, 0.90–10.32) compared with those aged 0–29 years [103].
Colorectal cancer	Patients with OSAS tend to have a higher risk of colorectal cancer (1.63 [1.12–2.38]) [102]. Another study has demonstrated similar results: after adjusting for potential confounders, patients with OSAS were associated with a significantly higher risk than those without OSAS (aHR, 1.80; 95% CI, 1.28–2.52). Moreover, the cumulative incidence of colorectal cancer was significantly higher in the OSAS cohort than in the comparison cohort [106].Nevertheless, several other studies have demonstrated a decreased risk of colorectal cancer [104].
Pancreatic cancer	Patients with OSAS tend to have an increased risk of pancreatic cancer (HR = 1.14, CI = 1.06–1.23) [106].
Kidney cancer	The risk of kidney cancer is debatable. Kendzerska and coworkers found no association between kidney cancer and OSAS [102]. Other studies have demonstrated an increased risk (HR = 1.30, CI = 1.23–1.37; SIR 2.24, 95% CI 1.82–2.72) [105].
Prostate cancer	One of the studies has demonstrated an increased risk of prostate cancer 1.63 (1.06–2.51) [102] while another demonstrated a protective effect (HR = 0.93, CI = 0.90–0.96 in both) [105].
Urinary cancer	Severe OSAS tends to increase urinary cancer 1.72 (1.08–2.75) [102].
Uterus	Uterus cancer is more frequent in OSAS patients (SIR 2.80, 95% CI 2.24–2.47) [104,105].

## Data Availability

Not applicable.

## References

[1] Pack A.I. (2006). Advances in sleep-disordered breathing. Am. J. Respir. Crit. Care Med..

[2] Corlateanu A., Covantev S., Botnaru V., Sircu V., Nenna R. (2017). To sleep, or not to sleep—That is the question, for polysomnography. Breathe.

[3] Bonsignore M.R., Baiamonte P., Mazzuca E., Castrogiovanni A., Marrone O. (2019). Obstructive sleep apnea and comorbidities: A dangerous liaison. Multidiscip. Respir. Med..

[4] Parati G., Lombardi C., Hedner J., Bonsignore M.R., Grote L., Tkacova R., Lévy P., Riha R., Bassetti C., Narkiewicz K. (2013). Recommendations for the management of patients with obstructive sleep apnoea and hypertension. Eur. Respir. J..

[5] Peng Y.-H., Liao W.-C., Chung W.-S., Muo C.-H., Chu C.-C., Liu C.-J., Kao C.-H. (2014). Association between obstructive sleep apnea and deep vein thrombosis/pulmonary embolism: A population-based retrospective cohort study. Thromb. Res..

[6] Emiley P. (2012). Sleep Apnea and Risk of Deep Vein Thrombosis: A Non-randomized, Pair-matched Cohort Study: Chou KT, Huang CC, Chen YM, et al. Am J Med 2012;125:374–80. J. Emerg. Med..

[7] Zozina V.I., Covantev S., Kukes V.G., Corlateanu A. (2021). Coenzyme Q10 in COPD: An Unexplored Opportunity?. COPD.

[8] Shamsuzzaman A.S.M., Gersh B.J., Somers V.K. (2003). Obstructive Sleep ApneaImplications for Cardiac and Vascular Disease. JAMA.

[9] Young T., Palta M., Dempsey J., Skatrud J., Weber S., Badr S. (1993). The Occurrence of Sleep-Disordered Breathing among Middle-Aged Adults. N. Engl. J. Med..

[10] Peppard P.E., Young T., Palta M., Skatrud J. (2000). Prospective Study of the Association between Sleep-Disordered Breathing and Hypertension. N. Engl. J. Med..

[11] Fletcher E.C., DeBehnke R.D., Lovoi M.S., Gorin A.B. (1985). Undiagnosed sleep apnea in patients with essential hypertension. Ann. Intern. Med..

[12] (1997). The Sixth Report of the Joint National Committee on Prevention, Detection, Evaluation, and Treatment of High Blood Pressure. JAMA Intern. Med..

[13] Chobanian A.V., Bakris G.L., Black H.R., Cushman W.C., Green L.A., Izzo J.J.L., Jones D.W., Materson B.J., Oparil S., Wright J.J.T. (2003). The Seventh Report of the Joint National Committee on Prevention, Detection, Evaluation, and Treatment of High Blood Pressure: The JNC 7 Report. JAMA.

[14] Whelton P.K., Williams B. (2018). The 2018 European Society of Cardiology/European Society of Hypertension and 2017 American College of Cardiology/American Heart Association Blood Pressure Guidelines: More Similar Than Different Comparison of the 2018 ESC/ESH and 2017 ACC/AHA Hypertension Guidelines Comparison of the 2018 ESC/ESH and 2017 ACC/AHA Hypertension Guidelines. JAMA.

[15] Narkiewicz K., Kato M., Phillips Bradley G., Pesek Catherine A., Davison Diane E., Somers Virend K. (1999). Nocturnal Continuous Positive Airway Pressure Decreases Daytime Sympathetic Traffic in Obstructive Sleep Apnea. Circulation.

[16] Marin-Oto M., Vicente E.E., Marin J.M. (2019). Long term management of obstructive sleep apnea and its comorbidities. Multidiscip. Respir. Med..

[17] Rizza S., Longo S., Piciucchi G., Romanello D., Mavilio M., Montagna M., Coppeta L., Martelli E., Magrini A., Federici M. (2020). Carotid intimal medial thickness in rotating night shift is related to IL1β/IL6 axis. Nutr. Metab. Cardiovasc. Dis..

[18] Shahar E., Whitney C.W., Redline S., Lee E.T., Newman A.B., Nieto F.J., O’Connor G.T., Boland L.L., Schwartz J.E., Samet J.M. (2001). Sleep-disordered Breathing and Cardiovascular Disease. Am. J. Respir. Crit. Care Med..

[19] Hanly P., Sasson Z., Zuberi N., Lunn K. (1993). ST-segment depression during sleep in obstructive sleep apnea. Am. J. Cardiol..

[20] Franklin K.A., Sahlin C., Nilsson J.B., Näslund U. (1995). Sleep apnoea and nocturnal angina. Lancet.

[21] Olafiranye O., Reis S., Strollo P.J. (2013). Sleep Apnea and Subclinical Myocardial Injury: Where Do We Stand?. Am. J. Respir. Crit. Care Med..

[22] Porto F., Sakamoto Y.S., Salles C. (2017). Association between Obstructive Sleep Apnea and Myocardial Infarction: A Systematic Review. Arq. Bras. Cardiol..

[23] Gottlieb Daniel J., Yenokyan G., Newman Anne B., O’Connor George T., Punjabi Naresh M., Quan Stuart F., Redline S., Resnick Helaine E., Tong Elisa K., Diener-West M. (2010). Prospective Study of Obstructive Sleep Apnea and Incident Coronary Heart Disease and Heart Failure. Circulation.

[24] Peker Y., Hedner J., Kraiczi H., Löth S. (2000). Respiratory Disturbance Index. Am. J. Respir. Crit. Care Med..

[25] Hersi A.S. (2010). Obstructive sleep apnea and cardiac arrhythmias. Ann. Thorac. Med..

[26] Becker H.F., Koehler U., Stammnitz A., Peter J. (1998). Heart block in patients with sleep apnoea. Thorax.

[27] Rossi V.A., Stradling J.R., Kohler M. (2013). Effects of obstructive sleep apnoea on heart rhythm. Eur. Respir. J..

[28] Hoffstein V., Mateika S. (1994). Cardiac arrhythmias, snoring, and sleep apnea. Chest.

[29] Djonlagic I., Guo M., Matteis P., Carusona A., Stickgold R., Malhotra A. (2014). Untreated sleep-disordered breathing: Links to aging-related decline in sleep-dependent memory consolidation. PLoS ONE.

[30] Emamian F., Khazaie H., Tahmasian M., Leschziner G.D., Morrell M.J., Hsiung G.-Y.R., Rosenzweig I., Sepehry A.A. (2016). The Association Between Obstructive Sleep Apnea and Alzheimer’s Disease: A Meta-Analysis Perspective. Front. Aging Neurosci..

[31] Andrade A.G., Bubu O.M., Varga A.W., Osorio R.S. (2018). The Relationship between Obstructive Sleep Apnea and Alzheimer’s Disease. J. Alzheimers Dis..

[32] Yeh N.C., Tien K.J., Yang C.M., Wang J.J., Weng S.F. (2016). Increased Risk of Parkinson’s Disease in Patients with Obstructive Sleep Apnea: A Population-Based, Propensity Score-Matched, Longitudinal Follow-Up Study. Medicine.

[33] Good D.C., Henkle J.Q., Gelber D., Welsh J., Verhulst S. (1996). Sleep-Disordered Breathing and Poor Functional Outcome After Stroke. Stroke.

[34] Netzer N., Werner P., Jochums I., Lehmann M., Strohl K.P. (1998). Blood Flow of the Middle Cerebral Artery with Sleep-Disordered Breathing. Stroke.

[35] Alexiev F., Brill A.-K., Ott S.R., Duss S., Schmidt M., Bassetti C.L. (2018). Sleep-disordered breathing and stroke: Chicken or egg?. J. Thorac. Dis..

[36] Yu K., Jiang Z.H., Zhang L.G. (2018). Therapeutic effects of long-term continuous positive airway pressure treatment on improving leptomeningeal collateral circulation in obstructive sleep apnea syndrome patients. Eur. Rev. Med. Pharmacol. Sci..

[37] Ifergane G., Ovanyan A., Toledano R., Goldbart A., Abu-Salame I., Tal A., Stavsky M., Novack V. (2016). Obstructive Sleep Apnea in Acute Stroke. Stroke.

[38] Tosun A., Köktürk O., Karata G.K., Çiftçi T.U., Sepici V. (2008). Obstructive sleep apnea in ischemic stroke patients. Clinics.

[39] Sharma S., Culebras A. (2016). Sleep apnoea and stroke. Stroke Vasc. Neurol..

[40] Odajiu I., Covantsev S., Sivapalan P., Mathioudakis A.G., Jensen J.S., Davidescu E.I., Chatzimavridou-Grigoriadou V., Corlateanu A. (2022). Peripheral neuropathy: A neglected cause of disability in COPD—A narrative review. Respir. Med..

[41] Mayer P., Dematteis M., Pépin J.L., Wuyam B., Veale D., Vila A., Lévy P. (1999). Peripheral neuropathy in sleep apnea. A tissue marker of the severity of nocturnal desaturation. Am. J. Respir. Crit. Care Med..

[42] Lüdemann P., Dziewas R., Sörös P., Happe S., Frese A. (2001). Axonal polyneuropathy in obstructive sleep apnoea. J. Neurol. Neurosurg. Psychiatry.

[43] Altintas N., Tutar U., Sariaydin M., Tiras R. (2013). A novel connection: Obstructive sleep apnoea and diabetic neuropathy. Eur. Respir. J..

[44] Dziewas R., Schilling M., Engel P., Boentert M., Hor H., Okegwo A., Lüdemann P., Ringelstein E.B., Young P. (2007). Treatment for obstructive sleep apnoea: Effect on peripheral nerve function. J. Neurol. Neurosurg. Psychiatry.

[45] Ejaz S.M., Khawaja I.S., Bhatia S., Hurwitz T.D. (2011). Obstructive sleep apnea and depression: A review. Innov. Clin. Neurosci..

[46] Shoib S., Malik J.A., Masoodi S. (2017). Depression as a Manifestation of Obstructive Sleep Apnea. J. Neurosci. Rural Pr..

[47] Kendzerska T., Gershon A.S., Hawker G.A., Tomlinson G.A., Leung R.S. (2017). Obstructive sleep apnoea is not a risk factor for incident hospitalised depression: A historical cohort study. Eur. Respir. J..

[48] Aloia M.S., Arnedt J.T., Smith L., Skrekas J., Stanchina M., Millman R.P. (2005). Examining the construct of depression in obstructive sleep apnea syndrome. Sleep Med..

[49] Schröder C.M., O’Hara R. (2005). Depression and Obstructive Sleep Apnea (OSA). Ann. Gen. Psychiatry.

[50] Garvey J.F., Pengo M.F., Drakatos P., Kent B.D. (2015). Epidemiological aspects of obstructive sleep apnea. J. Thorac. Dis..

[51] Baron K.G., Reid K.J., Kim T., Van Horn L., Attarian H., Wolfe L., Siddique J., Santostasi G., Zee P.C. (2017). Circadian timing and alignment in healthy adults: Associations with BMI, body fat, caloric intake and physical activity. Int. J. Obes..

[52] Antza C., Kostopoulos G., Mostafa S., Nirantharakumar K., Tahrani A. (2021). The links between sleep duration, obesity and type 2 diabetes mellitus. J. Endocrinol..

[53] Tan M.M.C., Jin X., Taylor C., Low A.K., Le Page P., Martin D., Li A., Joseph D., Kormas N. (2022). Long-Term Trajectories in Weight and Health Outcomes Following Multidisciplinary Publicly Funded Bariatric Surgery in Patients with Clinically Severe Obesity (≥3 Associated Comorbidities): A Nine-Year Prospective Cohort Study in Australia. J. Clin. Med..

[54] Taheri S., Lin L., Austin D., Young T., Mignot E. (2004). Short sleep duration is associated with reduced leptin, elevated ghrelin, and increased body mass index. PLoS Med..

[55] Khanijow V., Prakash P., Emsellem H.A., Borum M.L., Doman D.B. (2015). Sleep Dysfunction and Gastrointestinal Diseases. Gastroenterol. Hepatol..

[56] Li Q., Xu T., Shao C., Gao W., Wang M., Dong Y., Wang X., Lu F., Li D., Tan H. (2023). Obstructive sleep apnea is related to alterations in fecal microbiome and impaired intestinal barrier function. Sci. Rep..

[57] Koo D.L., Nam H. (2020). The Relationship between Obstructive Sleep Apnea and Functional Dyspepsia. J. Sleep Med..

[58] Hyun M.K., Baek Y., Lee S. (2019). Association between digestive symptoms and sleep disturbance: A cross-sectional community-based study. BMC Gastroenterol..

[59] Ghiasi F., Amra B., Sebghatollahi V., Azimian F. (2017). Association of irritable bowel syndrome and sleep apnea in patients referred to sleep laboratory. J. Res. Med. Sci..

[60] Mesarwi O.A., Loomba R., Malhotra A. (2019). Obstructive Sleep Apnea, Hypoxia, and Nonalcoholic Fatty Liver Disease. Am. J. Respir. Crit. Care Med..

[61] Petta S., Marrone O., Torres D., Buttacavoli M., Cammà C., Di Marco V., Licata A., Lo Bue A., Parrinello G., Pinto A. (2015). Obstructive Sleep Apnea Is Associated with Liver Damage and Atherosclerosis in Patients with Non-Alcoholic Fatty Liver Disease. PLoS ONE.

[62] Hirono H., Watanabe K., Hasegawa K., Kohno M., Terai S., Ohkoshi S. (2021). Impact of continuous positive airway pressure therapy for nonalcoholic fatty liver disease in patients with obstructive sleep apnea. World J. Clin. Cases.

[63] Chen L.D., Lin L., Zhang L.J., Zeng H.X., Wu Q.Y., Hu M.F., Xie J.J., Liu J.N. (2018). Effect of continuous positive airway pressure on liver enzymes in obstructive sleep apnea: A meta-analysis. Clin. Respir. J..

[64] Hobzova M., Ludka O., Stepanova R., Sova M., Sovova E. (2015). The positive effect of CPAP therapy on the level of liver enzymes in obstructive sleep apnea patients. Eur. Respir. J..

[65] Einhorn D., Stewart D.A., Erman M.K., Gordon N., Philis-Tsimikas A., Casal E. (2007). Prevalence of sleep apnea in a population of adults with type 2 diabetes mellitus. Endocr. Pract..

[66] West S.D., Nicoll D.J., Stradling J.R. (2006). Prevalence of obstructive sleep apnoea in men with type 2 diabetes. Thorax.

[67] Gottlieb D.J., Punjabi N.M., Newman A.B., Resnick H.E., Redline S., Baldwin C.M., Nieto F.J. (2005). Association of sleep time with diabetes mellitus and impaired glucose tolerance. Arch. Intern. Med..

[68] Rizza S., Luzi A., Mavilio M., Ballanti M., Massimi A., Porzio O., Magrini A., Hannemann J., Menghini R., Lehrke M. (2021). Alterations in Rev-ERBα/BMAL1 ratio and glycated hemoglobin in rotating shift workers: The EuRhythDia study. Acta Diabetol..

[69] Koren D., Levitt Katz L.E., Brar P.C., Gallagher P.R., Berkowitz R.I., Brooks L.J. (2011). Sleep architecture and glucose and insulin homeostasis in obese adolescents. Diabetes Care.

[70] Leproult R., Copinschi G., Buxton O., Van Cauter E. (1997). Sleep loss results in an elevation of cortisol levels the next evening. Sleep.

[71] Kelly A., Dougherty S., Cucchiara A., Marcus C.L., Brooks L.J. (2010). Catecholamines, adiponectin, and insulin resistance as measured by HOMA in children with obstructive sleep apnea. Sleep.

[72] Papanas N., Steiropoulos P., Nena E., Tzouvelekis A., Maltezos E., Trakada G., Bouros D. (2009). HbA1c is associated with severity of obstructive sleep apnea hypopnea syndrome in nondiabetic men. Vasc. Health Risk Manag..

[73] Tatti P., Strollo F., Passali D. (2013). Sleep apnea, sleep disturbance, and fasting glucose variability: A pilot study. J. Diabetes Sci. Technol..

[74] Yagihara F., Lucchesi L.M., D’Almeida V., de Mello M.T., Tufik S., Bittencourt L.R.A. (2012). Oxidative stress and quality of life in elderly patients with obstructive sleep apnea syndrome: Are there differences after six months of Continuous Positive Airway Pressure treatment?. Clinics.

[75] Yamauchi M., Nakano H., Maekawa J., Okamoto Y., Ohnishi Y., Suzuki T., Kimura H. (2005). Oxidative stress in obstructive sleep apnea. Chest.

[76] Lam J.C., Lam B., Yao T.J., Lai A.Y., Ooi C.G., Tam S., Lam K.S., Ip M.S. (2010). A randomised controlled trial of nasal continuous positive airway pressure on insulin sensitivity in obstructive sleep apnoea. Eur. Respir. J..

[77] Guest J.F., Panca M., Sladkevicius E., Taheri S., Stradling J. (2014). Clinical Outcomes and Cost-effectiveness of Continuous Positive Airway Pressure to Manage Obstructive Sleep Apnea in Patients with Type 2 Diabetes in the U.K. Diabetes Care.

[78] Xu P.H., Hui C.K.M., Lui M.M.S., Lam D.C.L., Fong D.Y.T., Ip M.S.M. (2019). Incident Type 2 Diabetes in OSA and Effect of CPAP Treatment: A Retrospective Clinic Cohort Study. Chest.

[79] West S.D., Nicoll D.J., Wallace T.M., Matthews D.R., Stradling J.R. (2007). Effect of CPAP on insulin resistance and HbA1c in men with obstructive sleep apnoea and type 2 diabetes. Thorax.

[80] Nicholl D.D.M., Ahmed S.B., Loewen A.H.S., Hemmelgarn B.R., Sola D.Y., Beecroft J.M., Turin T.C., Hanly P.J. (2012). Declining Kidney Function Increases the Prevalence of Sleep Apnea and Nocturnal Hypoxia. Chest.

[81] Hanly P.J. (2015). Consider the Kidney when Managing Obstructive Sleep Apnea. J. Clin. Sleep Med..

[82] Lin C.H., Perger E., Lyons O.D. (2018). Obstructive sleep apnea and chronic kidney disease. Curr. Opin. Pulm. Med..

[83] Marrone O., Bonsignore M.R. (2020). Sleep Apnea and the Kidney. Curr. Sleep Med. Rep..

[84] Lin C.-H., Lurie R.C., Lyons O.D. (2020). Sleep Apnea and Chronic Kidney Disease: A State-of-the-Art Review. Chest.

[85] Zamarrón E., Jaureguizar A., García-Sánchez A., Díaz-Cambriles T., Alonso-Fernández A., Lores V., Mediano O., Rodríguez-Rodríguez P., Cabello-Pelegrín S., Morales-Ruíz E. (2021). Obstructive sleep apnea is associated with impaired renal function in patients with diabetic kidney disease. Sci. Rep..

[86] Masuda T., Murata M., Honma S., Iwazu Y., Sasaki N., Ogura M., Onishi A., Ando Y., Muto S., Shimada K. (2011). Sleep-disordered breathing predicts cardiovascular events and mortality in hemodialysis patients. Nephrol. Dial. Transplant..

[87] Ozkok A., Kanbay A., Odabas A.R., Covic A., Kanbay M. (2014). Obstructive sleep apnea syndrome and chronic kidney disease: A new cardiorenal risk factor. Clin. Exp. Hypertens..

[88] Wingfield Digby J., Mathioudakis A., Heartshorne R., Mohammad M., Tewkesbury D., Needham M. (2017). CPAP appears to protect kidney function of patients with OSA. Eur. Respir. J..

[89] Beaudin A.E., Raneri J.K., Ahmed S.B., Hirsch Allen A.J.M., Nocon A., Gomes T., Gakwaya S., Series F., Kimoff J., Skomro R.P. (2021). Risk of chronic kidney disease in patients with obstructive sleep apnea. Sleep.

[90] Huang J., Song P., Hang K., Chen Z., Zhu Z., Zhang Y., Xu J., Qin J., Wang B., Qu W. (2021). Sleep Deprivation Disturbs Immune Surveillance and Promotes the Progression of Hepatocellular Carcinoma. Front. Immunol..

[91] Hakim F., Wang Y., Zhang S.X.L., Zheng J., Yolcu E.S., Carreras A., Khalyfa A., Shirwan H., Almendros I., Gozal D. (2014). Fragmented Sleep Accelerates Tumor Growth and Progression through Recruitment of Tumor-Associated Macrophages and TLR4 Signaling. Cancer Res..

[92] Lee D.-B., An S.-Y., Pyo S.-S., Kim J., Kim S.-W., Yoon D.-W. (2023). Sleep Fragmentation Accelerates Carcinogenesis in a Chemical-Induced Colon Cancer Model. Int. J. Mol. Sci..

[93] Rofstad E.K., Gaustad J.-V., Egeland T.A.M., Mathiesen B., Galappathi K. (2010). Tumors exposed to acute cyclic hypoxic stress show enhanced angiogenesis, perfusion and metastatic dissemination. Int. J. Cancer.

[94] Gozal D., Almendros I., Hakim F. (2014). Sleep apnea awakens cancer. OncoImmunology.

[95] Kontogianni K., Messini-Nikolaki N., Christou K., Gourgoulianis K., Tsilimigaki S., Piperakis S.M. (2007). DNA damage and repair capacity in lymphocytes from obstructive sleep apnea patients. Environ. Mol. Mutagen..

[96] McElroy J.A., Newcomb P.A., Titus-Ernstoff L., Trentham-Dietz A., Hampton J.M., Egan K.M. (2006). Duration of sleep and breast cancer risk in a large population-based case–control study. J. Sleep Res..

[97] Luo J., Sands M., Wactawski-Wende J., Song Y., Margolis K.L. (2013). Sleep disturbance and incidence of thyroid cancer in postmenopausal women the Women’s Health Initiative. Am. J. Epidemiol..

[98] Gozal D., Farré R., Nieto F.J. (2016). Obstructive sleep apnea and cancer: Epidemiologic links and theoretical biological constructs. Sleep Med. Rev..

[99] Campos-Rodriguez F., Martinez-Garcia M.A., Martinez M., Duran-Cantolla J., Peña Mde L., Masdeu M.J., Gonzalez M., Campo F., Gallego I., Marin J.M. (2013). Association between obstructive sleep apnea and cancer incidence in a large multicenter Spanish cohort. Am. J. Respir. Crit. Care Med..

[100] Marshall N.S., Wong K.K., Cullen S.R., Knuiman M.W., Grunstein R.R. (2014). Sleep apnea and 20-year follow-up for all-cause mortality, stroke, and cancer incidence and mortality in the Busselton Health Study cohort. J. Clin. Sleep Med..

[101] Chen J.C., Hwang J.H. (2014). Sleep apnea increased incidence of primary central nervous system cancers: A nationwide cohort study. Sleep Med..

[102] Kendzerska T., Povitz M., Leung R.S., Boulos M.I., McIsaac D.I., Murray B.J., Bryson G.L., Talarico R., Hilton J.F., Malhotra A. (2021). Obstructive Sleep Apnea and Incident Cancer: A Large Retrospective Multicenter Clinical Cohort Study. Cancer Epidemiol. Biomark. Prev..

[103] Chang W.P., Liu M.E., Chang W.C., Yang A.C., Ku Y.C., Pai J.T., Lin Y.W., Tsai S.J. (2014). Sleep apnea and the subsequent risk of breast cancer in women: A nationwide population-based cohort study. Sleep Med..

[104] Sillah A., Watson N.F., Schwartz S.M., Gozal D., Phipps A.I. (2018). Sleep apnea and subsequent cancer incidence. Cancer Causes Control.

[105] Gozal D., Ham S.A., Mokhlesi B. (2016). Sleep Apnea and Cancer: Analysis of a Nationwide Population Sample. Sleep.

[106] Chen C.Y., Hu J.M., Shen C.J., Chou Y.C., Tian Y.F., Chen Y.C., You S.L., Hung C.F., Lin T.C., Hsiao C.W. (2020). Increased incidence of colorectal cancer with obstructive sleep apnea: A nationwide population-based cohort study. Sleep Med..

